# Individually Tailored Internet-Based Treatment for Young Adults and Adults With Panic Attacks: Randomized Controlled Trial

**DOI:** 10.2196/jmir.1853

**Published:** 2012-06-26

**Authors:** Kristin Silfvernagel, Per Carlbring, Julia Kabo, Sara Edström, Jenny Eriksson, Lisa Månson, Gerhard Andersson

**Affiliations:** ^1^Department of Behavioural Sciences and LearningLinköping UniversityLinköpingSweden; ^2^Department of PsychologyUmeå UniversityUmeåSweden; ^3^Swedish Institute for Disability ReserachLinköping UniversityLinköpingSweden; ^4^Department of Clinical NeurosciencePsychiatry SectionKarolinska InstituteStockholmSweden

**Keywords:** Anxiety, depression, effectiveness, Internet-based treatment, cognitive behavior therapy

## Abstract

**Background:**

Previous studies on Internet-based treatment with minimal therapist guidance have shown promising results for several specific diagnoses.

**Objective:**

To (1) investigate the effects of a tailored, therapist-guided, Internet-based treatment for individuals with reoccurring panic attacks, and (2) to examine whether people in different age groups (18–30 years and 31–45 years) would respond differently to the treatment.

**Methods:**

We recruited 149 participants from an online list of individuals having expressed an interest in Internet treatment. Screening consisted of online questionnaires followed by a telephone interview. A total of 57 participants were included after a semistructured diagnostic interview, and they were randomly assigned to an 8-week treatment program (n = 29) or to a control condition (n = 28). Treatment consisted of individually prescribed cognitive behavior therapy text modules in conjunction with online therapist guidance. The control group consisted of people on a waitlist who later received treatment.

**Results:**

All dependent measures improved significantly immediately following treatment and at the 12-month follow-up. The between-group effect size on the primary outcome measure, the Panic Disorder Severity Scale, was *d *= 1.41 (95% confidence interval 0.81–1.95) at posttreatment. The within-group effect size from pretreatment to 12-month follow-up was *d *= 1.66 (95% confidence interval 1.14–2.35). Age group had no effect, suggesting that age did not influence the outcome.

**Conclusions:**

Tailoring an Internet-based treatment can be a feasible approach in the treatment of panic symptoms and comorbid anxiety and depressive symptoms. Younger adults benefit as much as adults over 30 years and up to 45 years of age.

**Trial Registration:**

Clinicaltrials.gov NCT01296321; http://www.clinicaltrials.gov/ct2/show/NCT01296321 (Archived by WebCite at http://www.webcitation.org/65wddsqlL)

## Introduction

Internet-based cognitive behavior therapy (iCBT) [1] has emerged as a novel evidence-based treatment for anxiety and mood disorders [2,3]. Guided iCBT has been found to be effective for numerous specific disorders such as panic disorder [4,5], generalized anxiety disorder [6,7], social anxiety disorder [8-10], posttraumatic stress disorder [11], specific phobia [12], major depression [3], stress [13], and somatic health problems such as irritable bowel syndrome [14]. One limitation of many previous iCBT trials on anxiety and depression is that they targeted specific disorders and that comorbid disorders could either be affected without being directly addressed or remain undetected and unchanged [15]. Another potential limitation of previous iCBT trials is that participants with comorbid anxiety and depression disorders often are excluded [16]. Structured or diagnosis-specific iCBT treatments are also limited in that they leave little room for clinician and patient preferences. One approach to iCBT aims to address these limitations by combining individually tailored treatment according to the participant’s needs and symptoms with transdiagnostic components. The idea is to address specific problems and comorbidities with the aim of increasing the scope of iCBT, and possibly increasing motivation and improving treatment outcome. Two previous trials have been conducted on tailored Internet-based treatment for anxiety disorders with comorbid anxiety and depression [17,18]. In this trial we further developed the protocol to treat symptoms of anxiety and depressive symptoms in the presence of panic attacks.

Panic attacks are common across psychiatric conditions, but far from all persons with panic attacks develop panic disorder [19]. Persons with recurrent panic attacks may, however, have other conditions that they want to address in treatment along with their panic symptoms, such as sleep problems, worry, low mood, and other symptoms. However, no trials have tested iCBT specifically for persons presenting with panic attacks regardless of the presence of a diagnosis such as panic disorder and social anxiety disorder. A few previous trials have been conducted on iCBT for younger persons with anxiety disorders. Two studies have investigated the effects of iCBT for social anxiety disorder in high school students [20] and in university students [21]. While these two studies found effects, adherence to treatment was poor. We therefore decided to target persons with panic symptoms with comorbid anxiety and depressive symptoms in this randomized controlled trial. We examined the effects of individually tailored iCBT for young adults aged 18–30 years and adults aged 31–45 years. We hypothesized that the treatment protocol would lead to reductions in symptom measures of panic, anxiety, and depression. We also expected increased quality of life for the participants in the treatment condition and that the treatment effects would remain at the 12-month follow-up. With regard to the two age groups, we did not expect differential outcomes.

## Methods

### Participants and Recruitment

**Figure 1:**
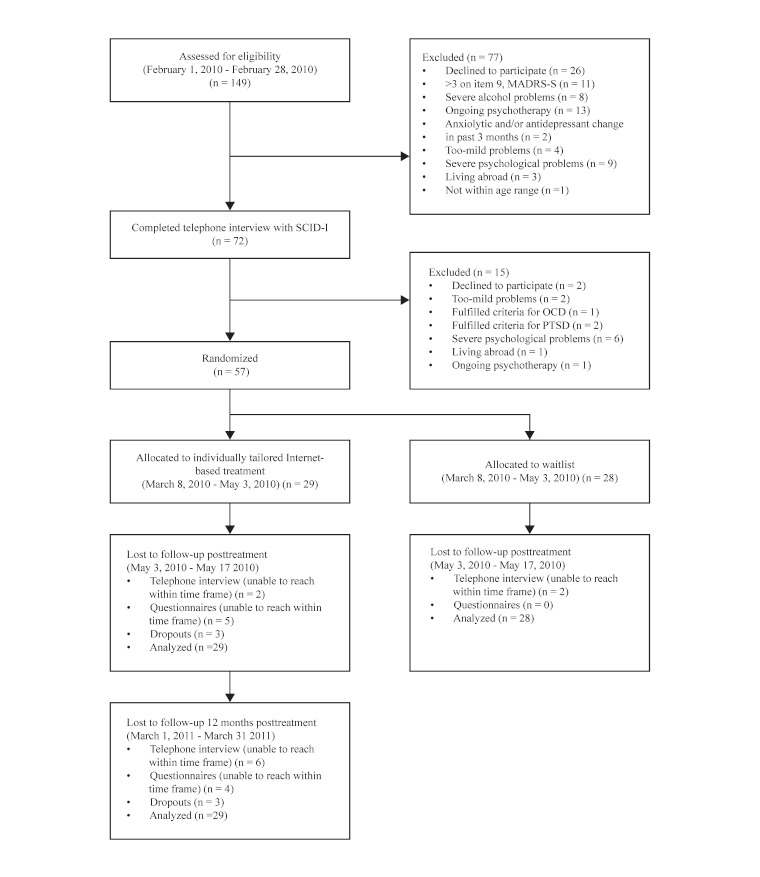
Flowchart of study participants, point of random allocation, and dropouts at each stage of the trial. MADRS-S = Montgomery-Åsberg Depression Scale; OCD = obsessive–compulsive disorder; PTSD = posttraumatic stress disorder; SCID-I = Structured Clinical Interview for DSM-IV Axis I Disorders.

We recruited participants via an online list (www.studie.nu) among individuals who had expressed an interest in participating in research on iCBT for panic disorder and generalized anxiety disorder via email. The list consisted of 1459 individuals who had been on the list for a minimum of 12,648 days, maximum 14,652 days, and mean of 13,298 days with a standard deviation of 505 days. They were presented with the project website, which contained information about the trial, how to register, and how to submit written informed consent. Screening consisted of the following questionnaires via the Internet: Montgomery-Åsberg Depression Scale-Self-rated (MADRS-S) [22]; Clinical Outcomes in Routine Evaluation-Outcome Measure (CORE-OM) [23]; Beck Anxiety Inventory (BAI) [24]; Quality of Life Inventory (QOLI) [25]; Alcohol Use Disorders Identification Test (AUDIT) [26]; and 13 additional questions with reference to demographic variables. If the participants met the initial inclusion criteria they underwent further screening consisting of the Structured Clinical Interview for DSM-IV Axis I Disorders (SCID-I) [27] and the Panic Disorder Severity Scale (PDSS) [28], conducted over the telephone by three clinical psychology MSc students who had completed their clinical training. The outcome measures used in the trial have been shown to have good psychometric properties when administered via the Internet [29], and the same accounts for administrating the SCID-I interview over telephone [30]. The principal investigator along with a psychiatrist further checked the SCID-I protocols and PDSS assessments for the participants before they enrolled in the trial. The participants had to have reoccurring panic attacks to be included. They could also fulfill the *Diagnostic and Statistical Manual of Mental Disorders*, 4th edition, text revision [27] criteria for any specific anxiety disorder, or anxiety disorder not otherwise specified except for obsessive–compulsive disorder and posttraumatic stress disorder, which would lead to exclusion. Participants could also meet the criteria for comorbid major depression, but not as the primary disorder. The participants had to be between the ages of 18 and 30 years or between 31 and 45 years; have a total score of <31 on the MADRS-S and a score <4 points on item 9 (suicidal thoughts) on MADRS-S; not currently be in psychotherapy; if on medication, be on stable dosage for the last 3 months; and not be at risk of alcohol abuse or fulfilling the criteria for current alcohol addiction. As outlined by the CONSORT flowchart (Figure 1), 149 individuals expressed an interest in the trial, which commenced in February 2010. After screening and diagnostic interview, we included 57 participants. For a demographic description of the participants, see Table 1. A separate demographic description for the different age groups is available on request.

**Table 1:** Demographic description of the participants at pretreatment.

		Treatment group (n = 29)	Control group (n = 28)	Total (n = 57)
**Gender, n (%)**				
	Male	8 (28%)	12 (43%)	20 (35%)
	Female	21 (72%)	16 (57%)	37 (65%)
**Age (years)**				
	Mean (SD)	32.3 (7.4)	32.5 (6.5)	32.4 (6.9)
	Minimum–maximum	20–45	21–44	20–45
	18–30	13 (45%)	12 (43%)	25 (44%)
	31–45	16 (55%)	16 (57%)	32 (56%)
**Marital status, n (%)**				
	Single without children	6 (21%)	6 (21%)	12 (21%)
	Single with children	1 (3%)	0 (0%)	1 (2%)
	Living apart without children	1 (3%)	2 (7%)	3 (5%)
	Living apart with children	1 (3%)	1 (4%)	2 (4%)
	Married/living together without children	5 (17%)	6 (21%)	11 (19%)
	Married/living together with children	15 (52%)	13 (46%)	28 (49%)
**Highest educational level, n(%)**				
	9-year compulsory school	2 (7%)	2 (7%)	4 (7%)
	Secondary school	11 (38%)	4 (14%)	15 (26%)
	Vocational (completed)	1 (3%)	3 (11%)	4 (7%)
	Collage/university (not completed	5 (17%)	7 (25%)	12 (21%)
	Collage/university (completed)	10 (35%)	12 (43%)	22 (39%)
**Psychotherapy, n (%)**				
	No experience	10 (35%)	6 (21%)	16 (28%)
	Previous experience	19 (66%)	22 (79%)	41 (72%)
**Medication, n (%)** ^a^				
	Ongoing	15 (52%)	12 (43%)	27 (47%)
	Completed	4 (14%)	5 (18%)	9 (16%)
	No experience	10 (35%)	11 (39)	21 (37%)
**Employment status, n (%)**				
	Self-employed	2 (7%)	0 (0%)	2 (4%)
	Employed	14 (48%)	14 (50%)	28 (49%)
	Unemployed	0 (0%)	4 (14%)	4 (7%)
	Student	5 (17%)	6 (21%)	11 (19%)
	On parental leave	4 (14%)	2 (7%)	6 (11%)
	Sick leave	4 (14%)	2 (7%)	6 (11%)

**SCID-I diagnosis, n (%)** ^b^				
	Panic disorder	2 (7%)	2 (7%)	4 (7%)
	Panic disorder + agoraphobia	24 (83%)	23 (82%)	47 (83%)
	Generalized anxiety disorder	2 (7%)	9 (32%)	11 (19%)
	Social phobia	1 (3%)	8 (29%)	9 (16%)
	Anxiety disorder not otherwise specified	1 (3%)	0 (0%)	1 (2%)
	Major depression	2 (7%)	3 (11%)	5 (9%)
	Comorbid disorders	5 (17%)	13 (46%)	18 (32%)

^a ^Anxiolytic and/or antidepressant. Needed to be stabilized for 3 months.

^b ^Structured Clinical Interview for DSM-IV Axis I Disorders.

### Treatment

The treatment consisted of 19 CBT modules derived from previous iCBT trials on panic disorder [16], generalized anxiety disorder, social phobia [31], depression [32], and tailored iCBT for anxiety [17,18] and depression (see Table 2 for prescribed modules). In this trial the first module (introduction) and the last module (relapse prevention) were fixed, and the following 17 were optional for the therapists to prescribe: cognitive restructuring (2 modules); panic disorder (2 modules); agoraphobia (1 module); generalized anxiety (3 modules); social anxiety (2 modules); behavioral activation (2 modules); applied relaxation (1 module); stress (1 module); mindfulness (1 module); problem solving (1 module); and insomnia (1 module). The modules are all based on established and evidence-based CBT components. The panic modules, for example, consisted of psychoeducation and interoceptive exposure. All modules included psychoeducation, nearly all contained exposure exercises, and some contained behavioral experiments depending on the content. All modules contained homework assignments for the participants, which consisted of questions on the psycheducational sections and tasks for the participant to complete, such as exposure exercises. The aim was to prescribe 6–8 modules within an 8-week time frame for each participant. A typical prescription for the participants could be an introduction, cognitive restructuring 1 and 2, panic disorder 1 and 2, agoraphobia, applied relaxation, and relapse prevention. Therapist guidance was included in the trial, since it has been found to improve outcomes when compared with most unguided treatments [33,34]. The therapists were three clinical psychology MSc students who had completed their clinical training and who were supervised by experienced clinical psychologists (senior authors). The therapists were responsible for 9–10 participants each during the 8 weeks of treatment. The treatment was individually tailored for each participant based on the results of the SCID-I interview and the clinical impression from the telephone interview. The participants were required to have access to a computer with an Internet connection and be able to download the prescribed modules in PDF format through an encrypted contact system, which they also used when communicating with their therapist. The participants were advised to spend 1 week on each prescribed module. If participants had not sent in their homework, which consisted of questions along with worksheets to report on their progress to their therapist within the time frame, their therapist would send out an email reminder. The therapists were instructed to check whether the participants had completed the assignments associated with each module and to provide individual feedback within 24 hours. This was scheduled to occur on Mondays. There were no automatic contacts; all contact was initiated by either the therapist or the participant. If the therapist judged that the participants had completed their homework, the therapist made the next module available through the encrypted contact handling system. Completion of the homework would mean that participants had answered questions about the treatment material and handed in descriptions of their interoceptive exposure, for example. If participants had not completed the previous module, the therapist would give instructions on what needed to be done to be able to move on to the next step. The therapists spent approximately 15 minutes per week per participant (estimated), which included administration as well as reading and responding to emails (estimated 19 emails between therapist and participant throughout the treatment) in the contact handling system.

**Table 2:** Number of prescribed treatment modules.

Module	Treatment group (n = 29)	Control group (n = 28)	Total (n = 57)
Introduction	29 (100%)	28 (100%)	57 (100%)
Cognitive restructuring 1	26 (90%)	22 (79%)	48 (84%)
Cognitive restructuring 2	26 (90%)	22 (79%)	48 (84%)
Panic 1	29 (100%)	28 (100%)	57 (100%)
Panic 2	29 (100%)	28 (100%)	57 (100%)
Agoraphobia	21 (72%)	22 (79%)	43 (75%)
Social anxiety 1	1 (3%)	3 (11%)	4 (7%)
Social anxiety 2	1 (3%)	3 (11%)	4 (7%)
Generalized anxiety 1	3 (10%)	6 (21%)	9 (16%)
Generalized anxiety 2	3 (10%)	6 (21%)	9 (16%)
Generalized anxiety 3	3 (10%)	6 (21%)	9 (16%)
Behavioral activation 1	0 (0%)	2 (7%)	2 (4%)
Behavioral activation 2	0 (0%)	2 (7%)	2 (4%)
Relaxation (applied)	15 (52%)	15 (54%)	30 (53%)
Sleep management	1 (3%)	1 (4%)	2 (4%)
Mindfulness	3 (10%)	2 (7%)	5 (9%)
Setting boundaries	3 (10%)	1 (4%)	4 (7%)
Solving problems	1 (3%)	0 (0%)	1 (2%)
Preventing relapse	29 (100%)	28 (100%)	57 (100%)

### Control Group

The control condition consisted of a waitlist group. Participants were informed that they would receive the treatment after 10 weeks when the treatment group had completed their treatment. They were also informed how to contact the study team if they had any questions during this time frame.

### Procedure and Design

We used a randomized controlled design to compare the effects of the treatment against waiting. The participants were divided into two groups so that the two predetermined age groups 18–30 years (young adults) and 31–45 years(adults) were equally represented in each condition. The blocked randomization process was conducted through an online true random number-generation service (random.org) independent of the investigators and therapists. The therapists in this trial were not aware of the participants’ age group. The project was approved by the regional ethics committee in Linköping and registered at ClinicalTrials.gov (NCT01296321). We obtained written informed consent through regular mail, which was sent to the study team. At posttreatment participants were instructed via email to complete the follow-up questionnaires and to participate in a semistructured telephone interview carried out by a blinded assessor who had no earlier contact with the participants. The same procedure was repeated at 12 months after treatment completion. We used the PDSS as the primary outcome measure in the trial.

### Statistical Analyses

To examine whether the randomization process had succeeded in generating a balanced distribution across the two conditions, we used independent *t *tests and the chi-square test for the demographic data and pretreatment measures. A mixed-models approach with an unstructured covariance structure was endorsed as a way to handle missing data at posttreatment and at the 1-year follow-up. As suggested by Gueorguieva and Krystal [35], we used mixed-effect models due to their advantages over traditional methods of repeated-measures analysis. We calculated between-group and within-group effect sizes (Cohen's *d*) from estimated means and observed pooled standard deviations. We also present a prespecified analysis of a 40% reduction from baseline on PDSS based on observed data, which is equivalent to the participant being much improved, according to the guidelines by Furukawa et al [36].

## Results

### Treatment Completion

Of the 29 participants in the treatment group, 7 (24%) completed all prescribed modules (6–8) within the 8-week treatment period. A total of 17 participants (59%) completed 50% of the prescribed modules and 14 (48%) completed 75% of the prescribed modules. The mean number of completed modules for the whole group was 5.0 (SD 2.6). The mean number of completed modules in the young adult group was 5.15 (SD 2.34) and the corresponding number in the adult group was 4.19 (SD 3.16). This difference was not statistically significant (*t*_27 _= 0.92, *P *= .37).

### Immediate Results: Treatment Versus Control

**Figure 2:**
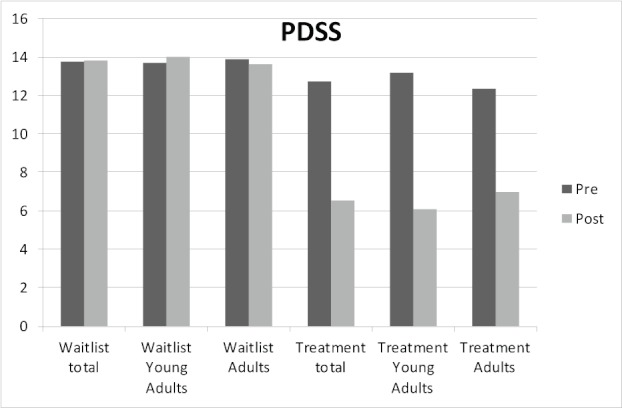
Change in observed mean scores in the Panic Disorder Severity Scale (PDSS) for the treatment and waitlist conditions and the two age groups pre- and posttreatment.

As is evident in Table 3, the treatment was superior to the control condition with significant interactions on all measures. A mixed-models analysis of the immediate results of the primary outcome measure, PDSS, showed a significant interaction (*F*_1,47.3 _= 29.6, *P *< .001, *d *= 1.41, *d*_young adult _= 1.59, *d*_adult _= 1.20; see Figure 2). For the secondary outcome measures CORE-OM (*F*_1,46.7 _= 10.8, *P *< .01, *d *= 1.01, *d*_young adult _= 1.24, *d*_adult _= 0.80), BAI (*F*_1,49.6 _= 4.6, *P *= .04, *d *= 0.57, *d*_young adult _= 0.93, *d*_adult _= 0.28), MADRS-S (*F*_1,47.5 _= 4.5, *P *= .04, *d *= 0.71, *d*_young adult _= 0.88, *d*_adult _= 0.60), and QOLI (*F*_1,48.8 _= 5.2, *P *= .03, *d *= 0.54, *d*_young adult _= 0.76, *d*_adult _= 0.32), we observed significant interaction effects and moderate to large between-group effect sizes. Age group did not interact with treatment condition across any measure. There was, however, an interaction of time and age group for BAI (*F*_1,49.6 _= 7.7, *P *< .01), which was caused by a slightly lower pretreatment value in the adult control group and thus not reflecting differential treatment effects.

**Table 3:** Immediate results with intention-to-treat analysis using mixed-effect model estimated means (ES) (n = 57), observed means (OM) (n = 50 for the main outcome measure^a ^and n = 49 for the secondary outcome measures), and standard deviation (observed) at pre- and posttreatment for the measures of panic, anxiety, depression, and quality of life.

Measure	Time	Group	Age group	ES	OM	SD	95% confidence interval
PDSS^a^	Pre	Treatment	Total	12.54	12.75	4.95	10.62–14.47
			Young adults	12.46	13.17	4.97	9.60–15.32
			Adults	12.63	12.33	5.12	10.05–15.21
		Control	Total	13.71	13.77	5.25	11.74–15.68
			Young adults	13.67	13.67	6.79	10.69–16.65
			Adults	13.75	13.86	3.74	11.17–16.33
	Post	Treatment	Total	6.39	6.54	4.97	4.30–8.48
			Young adults	5.57	6.08	4.03	2.55–8.60
			Adults	7.21	7.00	5.91	4.33–10.09
		Control	Total	13.79	13.81	5.49	11.71–15.85
			Young adults	14.00	14.00	6.58	10.92–17.09
			Adults	13.57	13.64	4.62	10.80–16.33
CORE-OM^b^	Pre	Treatment	Total	1.69	1.69	0.60	1.51–1.87
			Young adults	1.65	1.60	0.61	1.38–1.91
			Adults	1.73	1.78	0.59	1.49–1.97
		Control	Total	1.83	1.83	0.39	1.65–2.02
			Young adults	1.84	1.84	0.43	1.56–2.12
			Adults	1.83	1.82	0.37	1.59–2.07
	Post	Treatment	Total	1.14	1.13	0.56	0.92–1.36
			Young adults	0.96	0.93	0.44	0.65–1.27
			Adults	1.32	1.36	0.60	1.01–1.63
		Control	Total	1.69	1.69	0.53	1.49–1.89
			Young adults	1.65	1.65	0.68	1.35–1.96
			Adults	1.72	1.72	0.40	1.46–1.99
BAI^c^	Pre	Treatment	Total	28.20	28.43	10.06	24.94–31.46
			Young adults	29.15	28.36	11.64	24.31–34.00
			Adults	27.25	28.50	8.63	22.88–31.62
		Control	Total	27.97	27.50	8.91	24.63–31.31
			Young adults	31.25	31.25	10.15	26.21–36.29
			Adults	24.69	24.69	6.92	20.32–29.06
	Post	Treatment	Total	17.89	17.86	8.50	14.16–21.63
			Young adults	15.21	14.82	4.69	9.98–20.45
			Adults	20.58	21.20	10.60	15.25–25.90
		Control	Total	23.03	23.04	9.38	19.65–26.42
			Young adults	23.00	23.00	12.09	17.89–28.12
			Adults	23.06	23.06	7.16	18.63–27.49
MADRS-S^d^	Pre	Treatment	Total	16.24	15.81	7.35	13.38–19.11
			Young adults	15.92	15.00	8.05	11.67–20.18
			Adults	16.56	16.70	6.80	12.73–20.40
		Control	Total	18.13	17.93	8.38	15.20–21.05
			Young adults	19.50	19.50	7.48	15.07–23.93
			Adults	16.75	16.75	9.06	12.92–20.58
	Post	Treatment	Total	11.44	11.10	6.65	8.34–14.54
			Young adults	10.57	9.91	4.37	6.14–15.01
			Adults	12.30	12.40	8.57	7.96–16.64
		Control	Total	16.82	16.93	8.47	13.89–19.76
			Young adults	16.08	16.08	8.10	11.65–20.52
			Adults	17.56	17.56	8.94	13.72–21.40
QOLI^e^	Pre	Treatment	Total	0.83	0.96	1.92	0.18–1.48
			Young adults	0.93	1.08	2.44	–0.04–1.90
			Adults	0.72	0.83	1.25	–0.15–1.60
		Control	Total	0.74	0.76	1.68	0.08–1.41
			Young adults	0.64	0.64	1.90	–0.37–1.65
			Adults	0.84	0.84	1.55	–0.03–1.72
	Post	Treatment	Total	1.56	1.67	1.47	0.93–2.20
			Young adults	1.95	2.06	1.44	1.04–2.86
			Adults	1.17	1.24	1.46	0.28–2.05
		Control	Total	0.71	0.70	1.70	0.10–1.31
			Young adults	0.76	0.76	1.69	–0.15–1.68
			Adults	0.65	0.65	1.76	–0.14–1.44

^a ^Panic Disorder Severity Scale (PDSS).

^b ^Clinical Outcome in Routine Evaluation-Outcome Measure.

^c ^Beck Anxiety Inventory.

^d ^Montgomery-Åsberg Depression Rating Scale-Self-rated.

^e ^Quality of Life Inventory.

### Treatment Effects at 1-Year Follow-up

Participants in the treatment condition were contacted 1 year posttreatment and asked to complete the measures again in order to evaluate the long-term effects. A mixed-models analysis showed significant time effects for the primary outcome measure PDSS (*F*_1,18.3 _= 19.5, *P *< .001, estimated mean 4.80, SE 1.18, SD 4.66, *d* *= *1.66). We also found effects for the secondary outcome measures BAI (*F*_1,21.2 _= 15.2, *P *< .001, estimated mean 14.45, SE 2.02, SD 9.51, *d *= 1.45), MADRS-S (*F*_1,20.9 _= 6.4, *P *< .01, estimated mean 10.19, SE 1.97, SD 9.53, *d *= 0.75), and QOLI (*F*_1,20.3 _= 5.7, *P* *< *.01, estimated mean 1.94, SE 0.34, SD 1.55, *d *= 0.74). For these measures, the effect sizes were moderate to large. The exception was the CORE-OM (*F*_1,23.9 _= 9.7, *P *< .001, estimated mean 1.65, SE 0.10, SD 0.45, *d *= 0.07). There was no effect of age group for any of the measures.

### Clinical Significance

In the treatment group, 16 of the 24 (67%) participants who took part in the posttreatment telephone interview fulfilled the criteria of a 40% improvement on PDSS. The corresponding number in the control condition was 3 of 27 (11%) participants. Chi-square test showed a significant difference between the two conditions (χ^2^_1 _= 16.1, *P *< .001). At the 12-month follow-up, 14 of the 20 (70.0%) treated participants who took part in the telephone interview fulfilled the criteria of a 40% improvement on PDSS. Moreover, 16 of the 20 (80.0%) 20 participants no longer met the diagnostic criteria for the diagnosis they had at pretreatment.

## Discussion

The aim of this study was to investigate the effects of individually tailored iCBT for panic symptoms along with comorbid anxiety and depressive symptoms on young adults and adults. We found significant treatment effects for all dependent measures immediately following treatment and significant time effects at the 12-month follow-up, showing that a majority of the participants remained stable after completing their treatment. The between-group effect size on the primary outcome measure PDSS was *d *= 1.41 at posttreatment, and the within-group effect size was *d *=1.66 at the 12-month follow-up. The results of this trial, with moderate to large effect sizes, are consistent with previous trials of iCBT for panic disorder [16] and transdiagnostic iCBT treatment for anxiety [37], which shares some features with our treatment approach. The results of this trial, although focused on individuals with reoccurring panic attacks, are hence in line with previous trials of tailored iCBT for anxiety [17,18]. The participants did not have to meet all the diagnostic criteria for panic disorder to be included in the trial. However, most of them did, with 83% fulfilling the criteria for panic disorder with agoraphobia. This can be regarded as a limitation, as we aimed to target individuals with panic symptoms and not only persons with panic disorder. In light of the sample we recruited, we could expect results similar to those with iCBT for panic disorder. Regarding comorbid conditions, 32% had any comorbid disorder, which could have led to exclusion in previous trials on panic disorder. Previous iCBT trials have been criticized for setting too-strict inclusion criteria, thereby excluding participants with agoraphobia and comorbid disorders [38]. In this study we found that the treatment may work with less-stringent inclusion criteria.

We found no interaction effect between age group and the treatment condition over all measures; there was, however, an interaction between time and age group for the BAI. While this could indicate that the young adults as a group are more likely to improve spontaneously in the short time frame, it is more probable that this interaction is a chance finding with somewhat lower scores in the adult control group. The effect sizes across all measures showed a tendency for larger effects among the young adults but, due to the small sample size, this requires further investigation for any conclusions to be drawn. Perhaps most important in light of the problems with adherence reported in two previous iCBT trials on social anxiety disorder in high school students [20] and in university students [21] is that adherence was high in this trial, with no difference between the age groups.

This study has limitations. First, the prescription of treatment modules in the study may be unreliable because it was based on a structured diagnostic procedure (SCID-I) and clinical impression by relatively inexperienced clinicians. A more comprehensive clinical assessment, such as a functional analysis, may have resulted in the prescription of other modules. Worth mentioning is also that the treatment modules consisted of modules derived from diagnosis-specific trials and were mainly structured after each diagnosis (eg, panic disorder and not panic symptoms). We aim to address this in future trials on individually tailored treatment for anxiety symptoms, making the modules symptom specific and less difficult, or less specific for particular diagnoses. Second, the use of a waitlist control condition is a limitation of the present trial. The use of a passive control group means that the effect of nonspecific factors cannot be determined. The lack of a comparison group at the 12-month follow-up makes it impossible to conclude that the improvements over the follow-up period were due only to the effects of the individually tailored treatment. A third limitation is the size of the study, which in turn affects the generalizability of the results. The forth limitation is that the participants in this study had expressed an interest in iCBT for their problems and therefore the participants may have been highly motivated to undergo treatment. However, we did not measure how highly motivated the participants were. As a fifth limitation, the study was underpowered to detect age differences, and it is possible that small but clinically meaningful differences would have been detected with a larger sample. We do, however, note various clinical implications of the findings in the present trial. Individually tailored iCBT can broaden the use of iCBT to address comorbidity in anxiety disorder, allow for more flexibility, and emphasize the role of the clinician.

### Conclusions

The tentative conclusion drawn from these results is that tailoring iCBT can be a feasible approach in the treatment of panic symptoms, comorbid anxiety, and depressive symptoms. Future trials should directly compare individually tailored iCBT for anxiety versus traditional face-to-face CBT, preferably in a clinical setting with larger samples, along with examining how different age groups respond to this treatment format.

## Abbreviations

AUDIT: Alcohol Use Disorders Identification Test
BAI: Beck Anxiety Inventory
CORE-OM: Clinical Outcomes in Routine Evaluation-Outcome Measure
iCBT: Internet-based cognitive behavior therapy
MADRS-S: Montgomery-Åsberg Depression Scale-Self-rated
PDSS: Panic Disorder Severity Scale
QOLI: Quality of Life Inventory
SCID-I: Structured Clinical Interview for DSM-IV Axis I Disorders
